# Comparison of Upper Neck Loading in Young Adult and Elderly Volunteers During Low Speed Frontal Impacts

**DOI:** 10.3389/fbioe.2021.682974

**Published:** 2021-06-30

**Authors:** Carmen M. Vives-Torres, Manuel Valdano, Jesus R. Jimenez-Octavio, Julia Muehlbauer, Sylvia Schick, Steffen Peldschus, Francisco J. Lopez-Valdes

**Affiliations:** ^1^Instituto de Investigacion Tecnologica, ICAI, Engineering School, Universidad Pontificia Comillas, Madrid, Spain; ^2^Biomechanics and Accident Analysis, Ludwig Maximilians University (LMU), Munich, Germany

**Keywords:** frontal impact, head inertial properties, inverse dynamics, volunteer testing, occipital condyle loads

## Abstract

Cervical pain and injuries are a major health problem globally. Existing neck injury criteria are based on experimental studies that included sled tests performed with volunteers, post-mortem human surrogates and animals. However, none of these studies have addressed the differences between young adults and elderly volunteers to date. Thus, this work analyzed the estimated axial and shear forces, and the bending moment at the craniocervical junction of nine young volunteers (18–30 years old) and four elderly volunteers (>65 years old) in a low-speed frontal deceleration. Since the calculation of these loads required the use of the mass and moment of inertia of the volunteers' heads, this study proposed new methods to estimate the inertial properties of the head of the volunteers based on external measurements that reduced the error of previously published methods. The estimated mean peak axial force (Fz) was −164.38 ± 35.04 N in the young group and −170.62 ± 49.82 N in the elderly group. The average maximum shear force (Fx) was −224.42 ± 54.39 N and −232.41 ± 19.23 N in the young and elderly group, respectively. Last, the estimated peak bending moment (My) was 13.63 ± 1.09 Nm in the young group and 14.81 ± 1.36 Nm in the elderly group. The neck loads experienced by the elderly group were within the highest values in the present study. Nevertheless, for the group of volunteers included in this study, no substantial differences with age were observed.

## 1. Introduction

Neck injuries and pain are serious public health problems in the general population. The Global Burden of Diseases, Injuries, and Risk Factors Study 2017 estimated the point rate prevalence in 3551.1 cases and the number of years lived with disability associated to neck pain in 352 years per 100,000 population, globally (Safiri et al., 2020). The prevalence of neck pain has been reported to increase with age up to 70–74 years and then to decrease (Safiri et al., 2020). Motor vehicle crashes (MVC) are one of the main causes for neck injuries worldwide (Yadollahi et al., 2016; Umana et al., 2018). Although rare when compared to other injuries occurring in MVC, severe neck injuries can be life threatening or are associated with a high risk of severe impairment. A review of NASS-CDS data between 1994 and 2011 showed that spinal cord injury (SCI) occurred in one out of 1860 front seat occupants in tow-away crashes in the United States, with fracture-dislocation injuries occurring 5.3 times more often than SCI (Parenteau and Viano, 2014).

In frontal impacts, these injuries have been traditionally associated to the dynamic loading of the neck that occurs when the torso is suddenly stopped by the seat belt while the head continues pulling from the neck. As these loads cannot be measured directly without altering the tissue, the use of inverse dynamics methods has been proposed as a valid, non-invasive, method to estimate the craniocervical forces and moments experienced by volunteers and PMHS during frontal impacts (Funk et al., 2007; Lopez-Valdes et al., 2010; Seacrist et al., 2011; Beeman et al., 2016). However, this method requires calculating the mass and moment of inertia of the head, which cannot be directly measured when using human volunteers. In early studies with PMHS, the analysis of the head mass and moment of inertia involved the separation of the head from the neck (Walker et al., 1973). In more recent studies, less invasive methods, including the use of non-destructive computer models, have been used to accurately determine the human head anthropometry (Albery and Whitestone, 2003; Plaga et al., 2005; Damon, 2009). Other studies attempted to relate head inertial characteristics to external measurements (Clauser et al., 1969; McConville et al., 1980; Loyd et al., 2010; Seacrist et al., 2011). Such procedures have not been consistently used yet, requiring a more thorough investigation that could lead to the consolidation of a robust method to estimate such parameters.

As aforementioned, the prevention of MVC cervical injuries relies on monitoring the axial and shear forces and the bending moment measured at the upper and lower cervical spine of Anthropomorphic Test Devices (ATD) or dummies in simulated collisions to calculate neck injury indicators, such as the Neck Injury Criterion (Nij) that combines the axial force with the flexion/extension moment to predict the likelihood of cervical trauma (Li et al., 2019). These indices are then compared to corresponding thresholds that are based on previous experiments performed with Post Mortem Human Surrogates (PMHS), animals and live human volunteers (Mertz and Patrick, 1971; Prasad and Daniel, 1984), where severe injuries such as hemorrhages at the atlanto-occipital junction, cord transections, ligament and capsular partial and complete tears injuries were observed. The non-injury data obtained from these experiments have been used to propose the intercept values used in the development of the Nij injury criterion (Eppinger et al., 2000; Mertz et al., 2003). Despite the aforementioned experiments, cervical data from whole body experiments with volunteers in an automotive setup are still limited (Mertz and Patrick, 1971; Arbogast et al., 2009; Seacrist et al., 2011) and, only in a few cases, allow to study differences across different age groups which has focused mainly on understanding the differences between pediatric and adult subjects. Arbogast et al. (2009) found a decrease in the magnitude of flexion rotation with increasing age in the comparison between children (6–14 years old) and young adults (18–30 years old). For the same subjects, Seacrist et al. (2011) utilized inverse dynamics to estimate upper cervical neck loads and reported increasing bending moment and decreasing peak axial force with increasing age.

This work reviewed all the studies mentioned above and used the already available experimental data to propose a new method to calculate the head inertial properties of volunteers based on external measurements. This new method reduced considerably the error generated using the previously published methods. In addition, the axial and shear forces and the flexion moment at the craniocervical junction were estimated using inverse dynamics during a low-speed frontal deceleration of a set of volunteers. Two different volunteer age groups were analyzed: nine young adults (18–30 years old) and four elderly volunteers (>65 years old).

Thus, the current study had two objectives: to estimate the head mass and principal moment of inertia improving the mean error obtained in previous studies, and to verify whether there were age-related differences in the craniocervical loads in low-speed frontal impacts using inverse dynamics.

## 2. Materials and Methods

The experimental data used in this study were generated within the SENIORS project, funded by the European Union under the Horizon 2020 program (Grant agreement ID: 636136). The data used in this study correspond to low-speed frontal tests performed with volunteers from two different age groups.

A complete description of the test conditions, volunteers' characteristics, experimental data recorded during these tests and general kinematic and dynamic results can be downloaded from the website of the THUMS User Community (THUMSUserCommnunity, 2021).

### 2.1. Volunteer Characteristics and Procedures

Four elderly (>65 years old) and nine young (18–30 years old) male volunteers were recruited for the study. Subjects were chosen to be as close in height and weight as possible to the 50th male percentile (nominally: 175 cm, 78 kg). Volunteers reported not to have any health condition susceptible of being aggravated during the study. Prior to being exposed to the low-speed test, volunteers were measured and instrumented. Table 1 provides detailed information on the anthropometry of each of the test subjects.

**Table 1:** Anthropometry and main characteristics of volunteers.

**Subject ID**	**Age (years)**	**Stature (cm)**	**Weight (kg)**	**Neck girth (cm)**	**Head girth (cm)**	**Head breadth (cm)**	**Head depth (cm)**
Vol 01	18	171.0	75.5	38.0	59.5	15.3	19.4
Vol 02	18	176.5	77.7	36.5	57.0	15.9	19.9
Vol 03	21	179.5	73.0	37.0	59.0	15.7	20.1
Vol 04	21	179.0	79.4	37.0	58.0	15.5	19.9
Vol 05	22	167.0	75.3	38.5	55.0	14.4	19.2
Vol 07	71	176.5	99.0	46.0	60.0	16.3	20.5
Vol 08	82	165.3	78.2	41.5	57.0	16.9	19.3
Vol 09	67	169.0	88.2	44.5	59.5	15.8	20.3
Vol 10	28	172.0	68.4	37.5	56.0	14.8	20.0
Vol 11	70	172.5	89.6	41.0	58.0	16.0	20.0
Vol 12	25	174.0	73.0	38.0	59.5	17.0	22.0
Vol 13	26	174.0	64.6	37.0	57.0	15.0	20.0
Vol 14	21	173.0	86.7	43.0	61.0	15.5	22.0

The information sheet, informed consent, and the whole study procedure was reviewed and approved by CEICA (Ethical Commission for Clinical Research of Aragon), which was the official review board to ensure that the study was performed according to the required Ethical principles.

### 2.2. Calculation of Head Inertial Properties

The head mass and principal moment of inertia about the *y*-axis had to be estimated using anthropometric parameters that could be measured externally on the volunteers. The experimental data in Damon (2009), which included measurements of the head mass, moment of inertia and head dimensions from 100 PMHS (79 male and 21 female), were used to derive the estimations of the head inertial properties to be used in this study.

#### 2.2.1. Estimation of the Head Mass

Regression curves were generated for different potential predictors of the mass of the head (i.e. length, depth, width, circumference). The characteristic length, which is the sum of the head breadth, depth, and circumference, was also used. To test the accuracy of the estimations, the data in Damon (2009) were divided into a training set (80% of the data), and a test set (20% of the data).The normality of the residuals was studied, and the overall mean errors were calculated.

#### 2.2.2. Estimation of the Head Principal Moment of Inertia

Two regression models were analyzed including either head dimensions and the head mass as independent variables, or just head mass, due to the high correlation between the moment of inertia and the head mass reported in previous studies (Plaga et al., 2005). To test the accuracy of the estimations, the data in Damon (2009) were again divided into a training set (80% of the data) and a test set (20% of the data). Due to the high error obtained, a third approach was used in which the moment of inertia of the head was approximated by those of three-dimensional objects (ellipsoid and sphere) as shown in Equations (1, 2).

(1) $$\begin{array}{l} I_{Ellipsoid}\left(kg\;m^{2}\right)=\frac{1}{5}\cdot \left[Head\;mass\right]\cdot \left[a^{2}+b^{2}\right] \end{array}$$

(2) $$\begin{array}{l} I_{Sphere}\left(kg\;m^{2}\right)=\frac{2}{5}\cdot \left[Head\;mass\right]\cdot r^{2} \end{array}$$

The least-squares approach was used with combinations of the head depth, breadth, and circumference, to calculate the parameters needed (a, b, and r in Equations 1, 2).

### 2.3. Experimental Test Setup, Crash Pulse and Description of Tests

**Figure 1:**
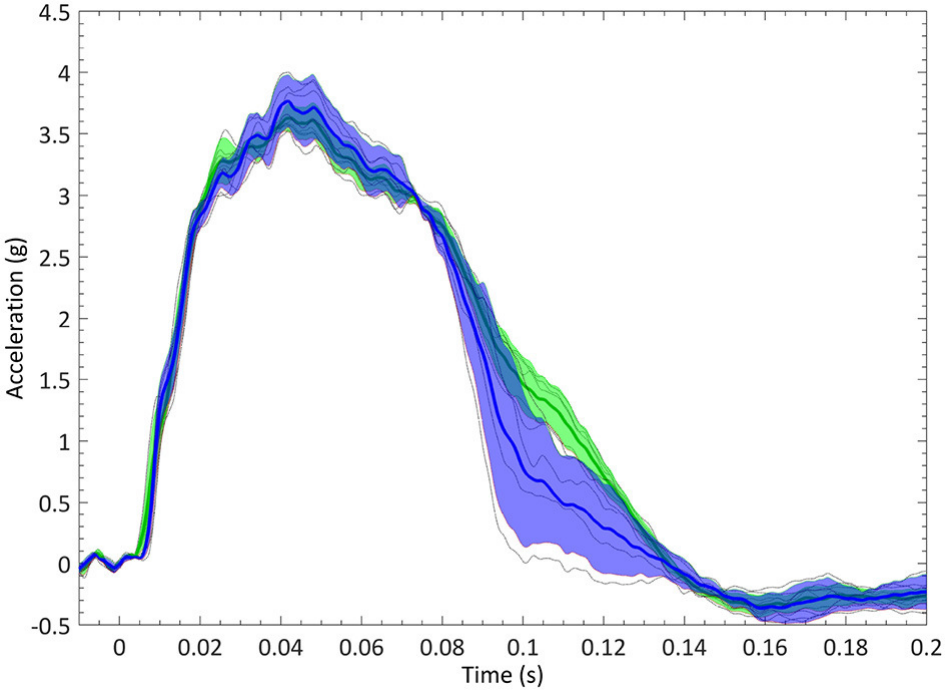
Sled deceleration pulse corridors in the young (blue) and elderly (green) groups. Solid lines are the average deceleration within the group. Shaded area corresponds to the one standard deviation corridor.

The experimental test fixture was designed to represent the seating posture of a passenger car occupant in a simplified manner. This test fixture had been used before in experiments simulating frontal crashes with other surrogates (the THOR dummy, PMHS tests) (Lopez-Valdes et al., 2018; Muehlbauer et al., 2019). The fixture consisted of a rigid seat, a rigid footrest, and a flexible backrest made out of three segments of metal wire. The seat geometry included several inclined plates in the rear-forward and mid-lateral directions, and was designed so that the pelvic sagittal displacement of the occupant in a frontal crash was similar to the one observed in a production car seat (Pipkorn et al., 2016b). Occupants were restrained by a non-retractor three-point seat belt. The position of the anchoring points of the seat belt was chosen based on previous studies to allow the comparison of the results (López-Valdés et al., 2016; Pipkorn et al., 2016a). The position of the footrest and of the seat belt D-ring were adjusted depending on each volunteer's anthropometry, but ensuring that the loading scenarios were dynamically similar. The magnitude and the time history of the sled deceleration were chosen based on previous studies to ensure a safe experimental environment for the volunteers (Arbogast et al., 2009; Lopez-Valdes et al., 2010). These previous studies had exposed volunteers to a triangular pulse with a peak of 3.5 g and a duration of 100 ms, and had reported that no volunteer had experienced pain or discomfort. The selected test pulse for this study is shown in Figure 1.

Each volunteer was exposed to a minimum of three tests, with the exception of volunteer 8, who participated only in two tests. A preliminary analysis of the data showed a different kinematic behavior between the first trial and the subsequent ones for each volunteer, which were more similar. Only the third trial of each volunteer was chosen for the inverse dynamics analyses. This gave the volunteers enough time to understand the testing procedure. Volunteers received an acoustic signal immediately before the start of the test and they were asked to remain relaxed. The second trial had to be used for volunteers 8 and 9, instead of the third one. For volunteer 9, the second test was chosen as the head band on which the forehead markers and sensors were placed moved with respect to the head in the third trial.

### 2.4. Experiments Instrumentation and Data Processing

**Figure 2:**
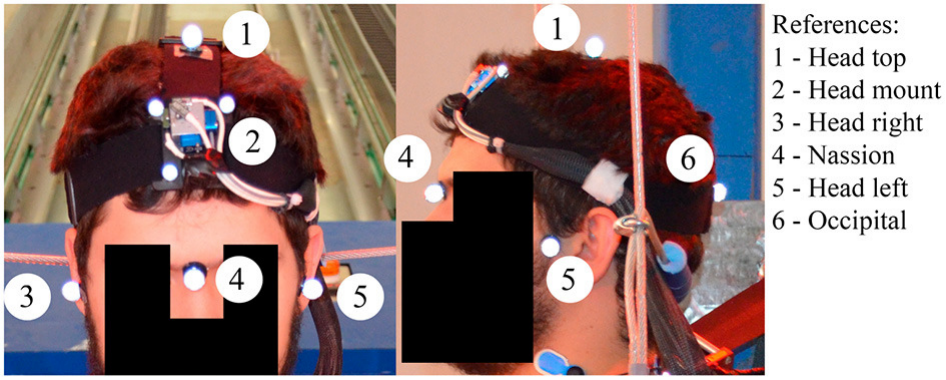
Detail of the 6 degree-of-freedom head cube and of the position of the sensors on the head of one of the volunteers.

A head mount that included a tridimensional accelerometer cube (Endevco 7264C, Meggitt, Irvine, US) and a tridimensional angular rate sensor (ARS PRO-18K, DTS, Seal Beach, US) was attached to an adjustable headband that was fastened around the head of the volunteers providing a secure fit to avoid any relative motion between the head and the instrumentation (Figure 2). All sensor data were recorded at 10,000 Hz using an external data acquisition system (PCI-6254, National Instruments; Austin, TX). Sensor data were filtered using a low pass filter with a cutoff frequency selected based on the characteristics of each of the signals to ensure that essential information was not removed in the filtering process. Table 2 shows the CFC class filters used in the analysis of each volunteer's data.

**Table 2:** Filters and cutoff frequencies used for each test.

**Subject ID**	**Head angular velocity**	**Head linear acceleration**
Vol 01	CFC 20	CFC 60
Vol 02	CFC 20	CFC 60
Vol 03	CFC 20	CFC 60
Vol 04	CFC 60	CFC 60
Vol 05	CFC 20	CFC 60
Vol 07	CFC 20	CFC 60
Vol 08	CFC 10	CFC 10
Vol 09	CFC 20	CFC 20
Vol 10	CFC 20	CFC 60
Vol 11	CFC 20	CFC 60
Vol 12	CFC 60	CFC 60
Vol 13	CFC 60	CFC 60
Vol 14	CFC 10	CFC 10

In addition to the above sensors, reflective markers were attached to selected anatomical landmarks on the volunteers, including: most lateral point of the Zygomatic bone (bilateral), Nasion and Opistocranion. Kinematic data were collected at 1,000 Hz using an optoelectric stereophotogrammetric system consisting of 10 cameras (Vicon, TS series, Oxford, UK). The system captured the position of the aforementioned retro-reflective spherical markers within a calibrated 3D volume. A calibration procedure, performed prior to testing, estimated the optical characteristics of each camera and established its position and orientation in a global coordinate system (GCS) that was fixed to the laboratory. The *x*-axis of the GCS pointed forward parallel to the moving direction of the sled, the *z*-axis pointed upwards and the *y*-axis was chosen to complete a right-hand coordinate system, resulting in a coordinate system in which the y and z axes pointed opposite to the SAE J211 recommendations. A photogrammetric algorithm within the Vicon Nexus software package (Nexus 1.8.5, Vicon, Oxford, UK) reconstructed the 3D position of each target for each video sample increment from the multiple 2D camera images.

### 2.5. Definition of Coordinate Systems

**Figure 3:**
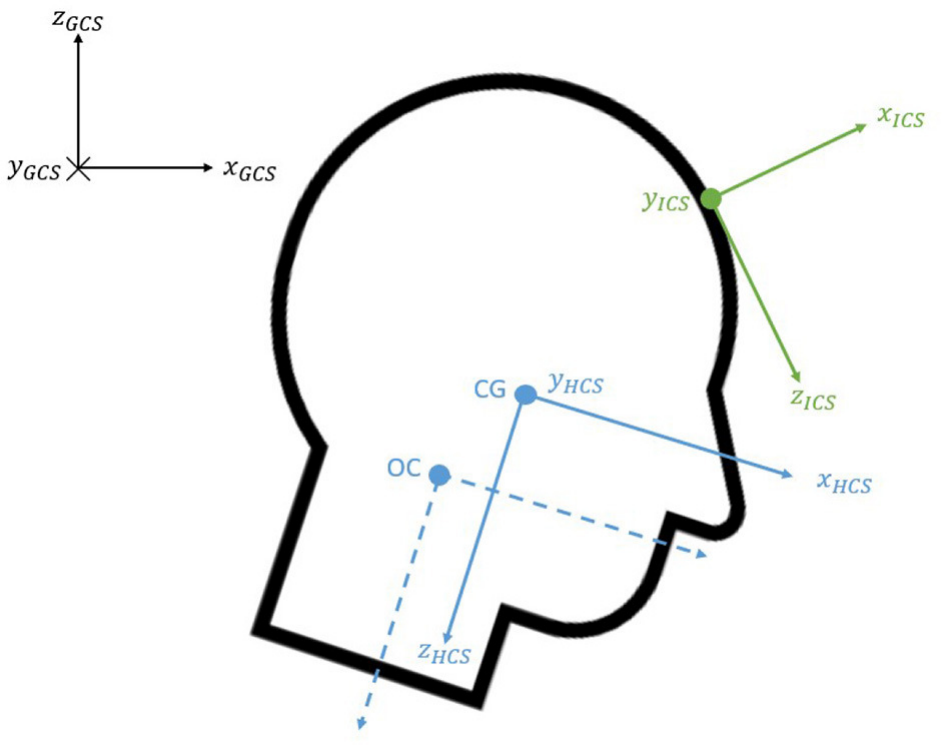
Coordinate systems.

Several coordinate systems were used in the study as illustrated in Figure 3. The position of the Vicon targets was expressed with respect to the fixed global coordinate system (GCS). The tridimensional accelerometer and the tridimensional angular rate sensor provided the corresponding data with respect to their local instrumentation coordinate system (ICS). The ICS was determined so that it would meet the criteria established in the SAE J211 standard. The origin was established at the point where the angular rate sensor was placed, which was estimated to be at the midpoint between two of the forehead markers.The polarity of the tridimensional angular rate sensor had been fixed so that the flexion motion was expressed according to the SAE J211 regulations.

The head anatomical coordinate system (HCS) was established at the center of gravity using the Frankfort plane and head anatomical landmarks (Beier et al., 1980; Albery and Whitestone, 2003; Plaga et al., 2005). The *y*-axis was the vector joining both tragions; the *x*-axis was perpendicular to this vector and passed through the midpoint of the infraorbitals pointing forward; the *z*-axis completed the standard orientated coordinate system and pointed downwards (SAE, 2007). Like previous studies (Lopez-Valdes et al., 2010; Seacrist et al., 2011), a coordinate system, parallel to the HCS and located at the Occipital-Condyle joint (OC), was used to express the upper neck loads.

### 2.6. Upper Neck Loading

Upper neck loads were estimated for the volunteers during low speed frontal sled tests using inverse dynamics. The analysis was performed only in the sagittal plane, as the out-of-plane motion was shown to be negligible by calculating the angle formed by the instrumentation *y*-axis with the global *y*-axis (the misalignment between these two vectors was found to be under 2%).

The initial angle of the ICS about the global *y*-axis was estimated using the dot product between the ICS *x*-axis unit vector and the GCS *x*-axis unit vector (Equation 3). The initial head angle was calculated as the angle formed by the marker placed at the top of the head and the head center of gravity (Equation 4). The head center of gravity was computed to be at the midpoint between the markers located at either side of the head.

(3) $$\theta_{Instrum}(t=0)=\arccos({\underline{X}}_{ICS}\cdot {\underline{X}}_{GCS})$$

(4) $$\begin{array}{l} \theta_{Head}\left(t=0\right)=-\arctan\left(\frac{X_{Head_{T}}-X_{Head_{CG}}}{Z_{Head_{T}}-Z_{Head_{CG}}}\right) \end{array}$$

Once the initial values of these angles were known, the angles formed by the ICS and the HCS at any other instant in time were obtained through integration of the angular velocity time-history. The HCS was then determined according to the SAE J211-based ATD coordinate system. To double-check the negligibility of the out-of-plane motion, the deviation between the HCS *y*-axis and the GCS *y*-axis was computed again at each time step and was found to be minimal. As the forces and moments applied at the craniocervical joint were to be expressed with respect to the HCS, the necessary rotation matrices to transform the variables to the HCS were calculated.

The angular velocity was differentiated to obtain the angular acceleration of the head, with an initial acceleration of zero. A CFC 60 filter was used to eliminate high frequency components that could have been introduced in the differentiation of the angular velocity, as suggested in previous publications (Lopez-Valdes et al., 2010; Seacrist et al., 2011).

The linear acceleration at the center of gravity of the head was computed using the existing kinematic relationship between the acceleration of two points belonging to the same rigid body as shown in Equation (5), where *a*_*CG*_ is the linear acceleration at the center of gravity of the head; *a*_*Instrum*_ is the linear acceleration measured using the tri-axial accelerometer at the origin of the ICS; and ρ is the vector from the origin of the ICS to the center of gravity of the head, which was calculated at *t* = 0 and then rotated according to the motion of the head.

(5) $${\underline{a}}_{CG}={\underline{a}}_{Instrum}+{\underline{\ddot{\theta }}}_{Head}\times \underline{\rho }+{\underline{\dot{\theta }}}_{Head}\times ({\underline{\dot{\theta }}}_{Head}\times \underline{\rho })$$

**Figure 4:**
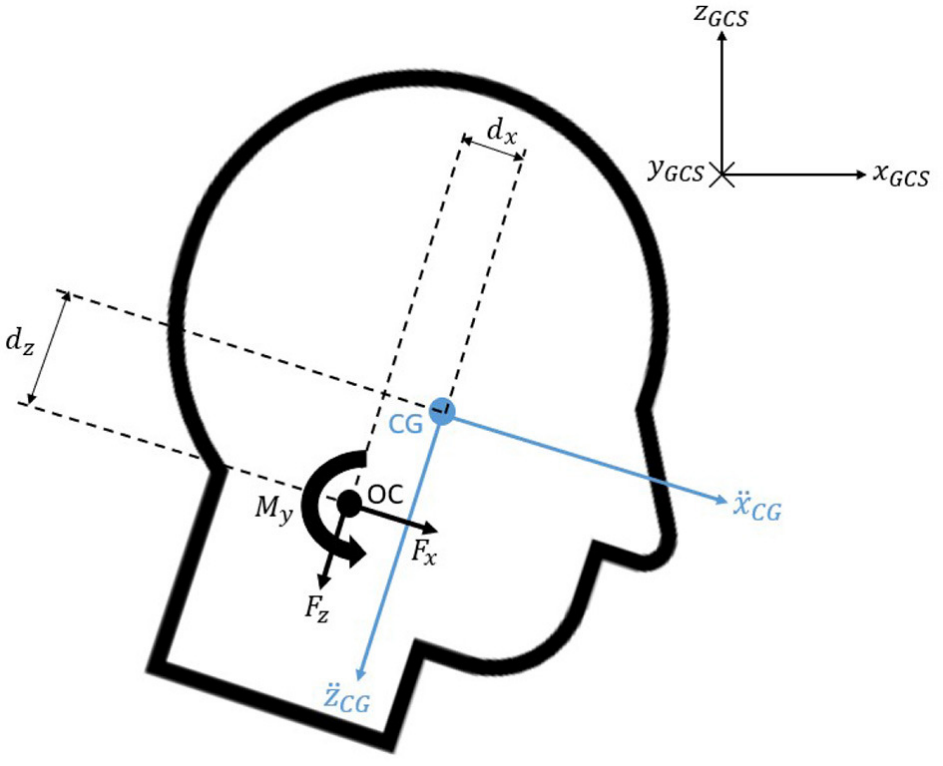
Free body diagram of the head.

Then, the craniocervical forces and moment were calculated as shown in Equations (6, 7), where the head mass and the moment of inertia (*I*_*Head*_) for each volunteer had been determined as aforementioned; (ẍ) and ($\ddot{z}$) were the x and z components of the linear accelerations at the center of gravity of the head; and (*d*_*x*_) and (*d*_*z*_) represented the distances between the center of gravity of the head and the occipital condyle joint in the x and z direction with respect to the HCS. Figure 4 illustrates the position and positive polarity of the estimated neck loads.

(6) $$\begin{array}{l} \sum \underline{F}=\left[\begin{array}{c} F_{x}\\ F_{z} \end{array}\right]=\left[Head\;mass\right]\cdot {\left[\begin{array}{c} ẍ\\ \ddot{z} \end{array}\right]}_{CG}-\left[Head\;mass\right]\cdot g\cdot \left[\begin{array}{c} \sin\theta_{Head}\\ \cos\theta_{Head} \end{array}\right] \end{array}$$

(7) $$\begin{array}{l} M_{y}=I_{Head}\cdot {\ddot{\theta }}_{Head}-F_{x}\cdot \;d_{z}-F_{z}\cdot \;d_{x} \end{array}$$

The distances between the center of gravity and the occipital condyle joint (*d*_*x*_ and *d*_*z*_) were calculated for each subject according to their head depth and height, respectively, as suggested in previous research (Seacrist et al., 2011). This is shown in Equation (8).

(8) $$\left\{\begin{array}{c} d_{x}=0.102\cdot H_{Depth}\\ d_{z}=0.260\cdot H_{Height} \end{array}\right\}$$

## 3. Results

None of the volunteers experienced any cervical pain or discomfort, nor any other symptoms (headache, back pain, etc.), that could be associated to the tests.

### 3.1. Head Inertial Properties

As aforementioned, the experimental data in Damon (2009) were used to calculate the mean errors in the estimation of the inertial properties of the head of 100 PMHS using previous methods reported in the literature and the new relations proposed in the current study.

#### 3.1.1. Head Mass

Two regression models were built to estimate the mass head of the 20% PMHS data in Damon (2009) used as validation data. The regression model that yielded the lowest error was the one shown in Equation (9), that used the characteristic length (CL) as the single predictor of head mass. Table 3 shows the mean errors obtained in the estimation of the head mass using the method developed here and comparing it to the estimations obtained with the methods suggested in previous research. The same set of data taken from Damon (2009) was used for the comparison of the error between the different methods.

(9) $$\begin{array}{l} Head\;mass\left(kg\right)=4.4655\cdot \left[CL\left(m\right)\right] \end{array}$$

**Table 3:** Mean errors and standard deviations obtained in the estimation of the head inertial properties.

	**Present study**			**Seacrist et al., 2011**
	**Training set (80%)**	**Validation set (20%)**	**Overall (100%)**	**Overall (100%)**
Head mass	11.00, 9.39%	12.78, 7.68%	11.36, 9.07%	18.16, 19.61%
I_*yy*_	7.15, 6.85%	8.30, 5.62%	13.93, 12.38%	27.89, 31.19%

The overall mean error using the procedure developed here was 11.36 ± 9.07%, which was below the error obtained in previous studies. Thus, this method was applied to estimate the mass of the head of the volunteers included in this study. The calculated values are shown in Table 4. The average and standard deviation head mass for the volunteer group was 4.20 ± 0.13 kg.

**Table 4:** Calculated head inertial properties.

**Subject ID**	**Head mass (kg)**	**I_***yy***_ (kg m^**2**^)**
Vol 01	4.21	0.0232
Vol 02	4.14	0.0242
Vol 03	4.23	0.0250
Vol 04	4.17	0.0241
Vol 05	3.96	0.0211
Vol 07	4.32	0.0267
Vol 08	4.16	0.0235
Vol 09	4.27	0.0257
Vol 10	4.05	0.0234
Vol 11	4.20	0.0247
Vol 12	4.40	0.0310
Vol 13	4.11	0.0238
Vol 14	4.40	0.0303

#### 3.1.2. Head Moment of Inertia

As with the estimation of the head mass, the data in Damon (2009) was used to compare the accuracy of the estimation of the head moment of inertia using relationships available in the literature. As the error in these methods was high, alternative methods based on linear regression and in the approximation of the shape of the head by two 3D volumes of known moment of inertia were developed. These methods used 80% of the data in Damon (2009) as a training set. The relationship that found the minimum error between the newly ones proposed was the one based on considering the head as a 3D ellipsoid, as shown in Equation (10).

(10) $$\begin{array}{l} I_{yy}(kg\;m^{2})=\frac{1}{5}\cdot [Head\;mass(kg)]\cdot [{\left(0.7922\cdot [H_{Depth}(m)]\right)}^{2}\\ \;+{\left(0.4124\cdot [H_{Breadth}(m)]\right)}^{2}] \end{array}$$

This relationship proposed in Equation (10) resulted in a mean 13.93 ± 12.38% error that was substantially smaller than the one obtained using previously published methods. Even if the standard deviation (SD) obtained with the newly proposed methodology was almost as high as the mean error, this SD was also smaller than the one obtained with the existing published methods (see Table 3). Thus, the moment of inertia of the head of the volunteers of the study were calculated using this procedure and are shown in Table 4. The average and standard deviation of the head principal moment of inertia for the volunteer group was 0.0251 ± 0.0028 kg m^2^.

### 3.2. Upper Neck Loading

**Figure 5:**
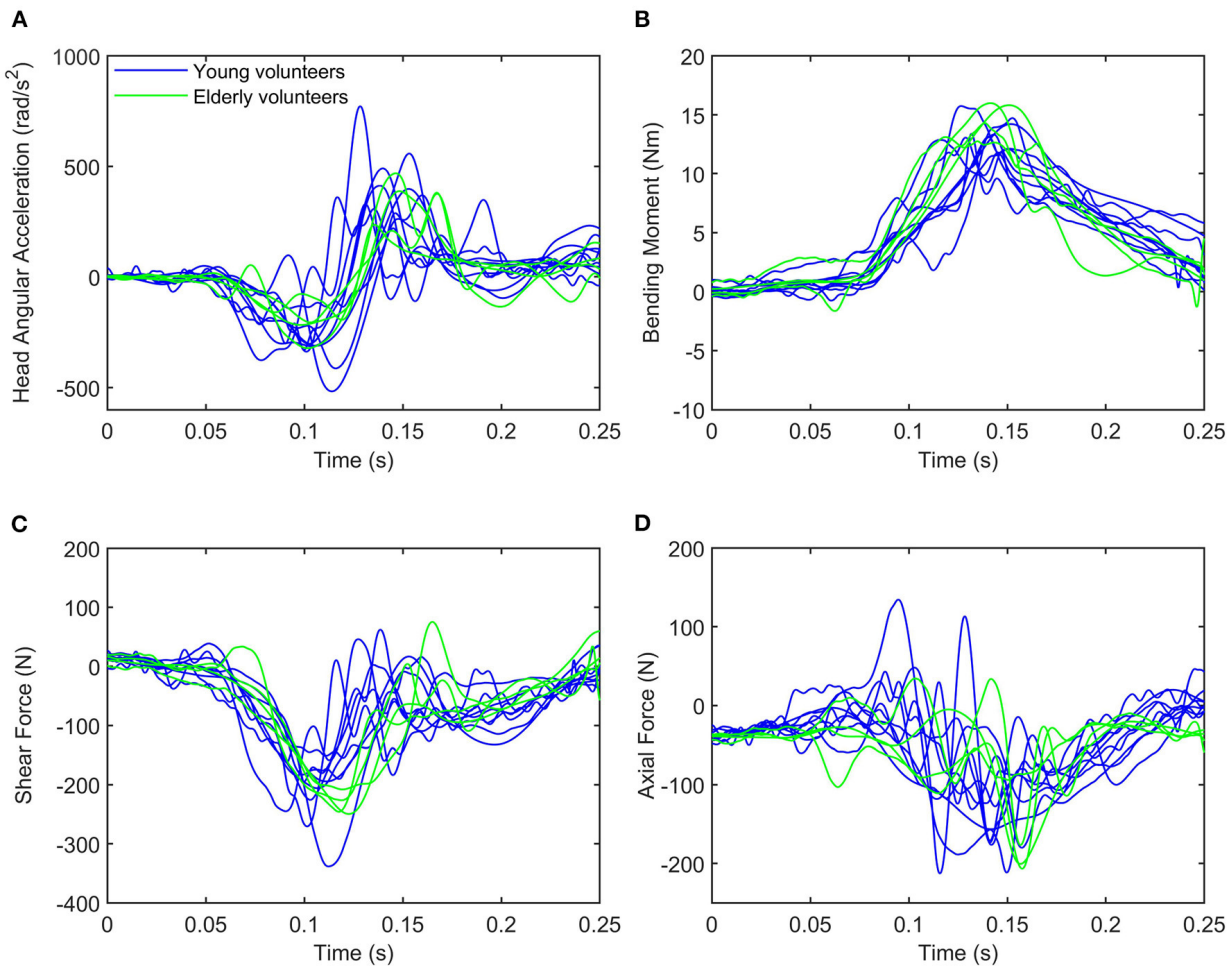
Time history of **(A)** head angular acceleration, **(B)** bending moment, **(C)** shear force, and **(D)** axial force. The blue lines represent upper neck loading and head angular acceleration for young volunteers, while the green lines are used for elderly volunteers.

The time history plots obtained for the estimation of the shear and axial forces, bending moment, and head angular acceleration for each volunteer are shown in Figure 5. Green solid traces show the results obtained for the volunteers in the elderly group, while blue solid ones correspond to the volunteers in the younger age group.

#### 3.2.1. Head Angular Acceleration

The calculated head angular acceleration is shown in subplot A in Figure 5. The time history plot shows that the head is accelerated in the negative HCS *y*-axis up to approximately 100 ms (flexion) and then it accelerates in the opposite direction (extension) up to 200 ms. This trend is common to all volunteers regardless of the group age. Vol 02 and 05, both within the younger group, exhibited peak flexion values that were considerably greater than those of the other volunteers regardless of the age group (−412.96 and −516.88 rad/s^2^). Vol 02 also sustained one of the two highest values in the angular acceleration in extension, although the peak value was observed in the results of Vol 01 (772.83 rad/s^2^). Vol 04 exhibited the minimum value in the flexion motion for both age groups (−142.70 rad/s^2^). With the exception of this subject, three out of the four elderly volunteers showed smaller peak values in flexion than any volunteer in the younger group. Vol 11 in the elderly group sustained a similar angular acceleration value than the ones observed in the younger group. The peak values of the head angular acceleration are shown in Table 5.

**Table 5:** Peak head angular acceleration.

**Subject ID**	${\ddot{\theta }}_{min}$ **(rad/s^**2**^)**	**Time (s)**	${\ddot{\theta }}_{max}$ **(rad/s^**2**^)**	**Time (s)**
**Young volunteers**				
Vol 01	–377.58	0.08	772.83	0.13
Vol 02	–412.96	0.12	558.83	0.15
Vol 03	–313.92	0.10	491.60	0.14
Vol 04	–142.70	0.09	288.12	0.15
Vol 05	–516.88	0.11	368.80	0.16
Vol 10	–303.45	0.10	412.24	0.14
Vol 12	–338.83	0.10	360.37	0.12
Vol 13	–331.98	0.10	307.35	0.14
Vol 14	–311.98	0.11	397.17	0.15
**Elderly volunteers**				
Vol 07	–194.72	0.12	235.19	0.14
Vol 08	–217.80	0.10	387.13	0.15
Vol 09	–206.42	0.10	375.19	0.17
Vol 11	–322.88	0.10	467.98	0.15

#### 3.2.2. Shear and Axial Force, and Moment at the Craniocervical Junction

Time history plots of the forces and moment estimated at the craniocervical junction are shown in Figures 5B–D.

All volunteers exhibited a similar behavior regarding the time history of the shear neck force, showing a negative peak (indicating that the neck pulls from the head as the head moves forward) at around 100 ms. There was less variability in the timing within the elderly volunteer group (in which the peak shear force was always obtained at *t* = 120 ms) than within the younger volunteer group (in which the time of the peak force ranged between 90 and 120 ms). As for the magnitude, Vol 05 exhibited the largest shear force (−338.32 N) and Vol 04 the lowest shear force peak value (−154 N) observed for any of the volunteers. These observations coincide with the ones discussed above related to the angular acceleration and it is probably an indication of the link between the value of the shear force and the rotational acceleration of the head. The range of peak shear forces was greater for the young adults than for the elders, being (−338.32 N, −190.28 N) and (−207.97 N, −249.54 N), respectively. The peak values of the shear force estimated for all the volunteers are included in Table 6.

**Table 6:** Peak upper neck loads.

**Subject ID**	**F_***x***_ (N)**	**Time (s)**	**F_***z***_ (N)**	**Time (s)**	**M_***y***_ (Nm)**	**Time (s)**
**Young volunteers**						
Vol 01	–244.97	0.09	–212.82	0.12	13.08	0.13
Vol 02	–190.28	0.12	–127.67	0.14	14.73	0.15
Vol 03	–205.83	0.11	–122.00	0.16	13.34	0.14
Vol 04	–154.82	0.12	–123.68	0.14	12.12	0.15
Vol 05	–338.32	0.11	–211.66	0.15	12.88	0.12
Vol 10	–225.80	0.10	–180.16	0.16	13.41	0.13
Vol 12	–270.61	0.10	–171.29	0.14	15.75	0.13
Vol 13	–194.72	0.10	–173.55	0.14	13.16	0.14
Vol 14	–194.45	0.11	–156.56	0.14	14.22	0.15
**Elderly volunteers**						
Vol 07	–245.85	0.12	–98.44	0.12	13.12	0.12
Vol 08	–249.54	0.12	–200.57	0.16	15.82	0.15
Vol 09	–207.97	0.12	–206.70	0.16	15.99	0.14
Vol 11	–226.29	0.12	–176.78	0.16	14.31	0.14

More variability could be observed in the results for the estimation of the neck axial force as shown in Figure 5. In particular, Vol 01 exhibited a different behavior than any of the other volunteers regardless of the age group: with a positive compression force observed at around *t* = 90 ms and the largest peak tension force (−212.82 N) obtained at *t* = 120 ms. No other volunteers exhibited this phase change. In general, volunteers sustained a peak tension force delayed some 30 ms from the peak shear force. This behavior indicates that the peak tension force occurs when the head has reached its maximum forward excursion and undergoes a flexion motion that will attempt to elongate the neck. Peak axial forces ranged between (−212.82 N, −122.00 N) in the young volunteer group and between (−206.70 N, −98.44 N) in the elderly volunteer group. These values are shown in Table 6.

The timing for the maximum moment My was more similar to the one of the peak tension force than to the one in which the peak shear force was observed. It is again linked to the fact that the flexion motion of the head starts only when the forward motion has finished. This timing of the peak flexion moment is very similar to the peak of the positive head angular acceleration discussed above. The time history plot of the My moment shown in Figure 5 shows that there were no substantial differences neither in the magnitude nor in the phasing between the two age groups. Peak values of the My moment are included in Table 6 and ranged between (12.12 Nm, 15.99 Nm).

## 4. Discussion

This is the first study that reports axial and shear forces, and flexion moment at the atlanto-occipital junction of young and elderly volunteers using inverse dynamics. The current study complements the data presented by Seacrist et al. (2011) that included a comparison of the same upper cervical loads but between pediatric and young adult volunteers.

The craniocervical junction consists of two joints: the atlanto-occipital and the atlanto-axial. While the joint mechanics of the first one are determined by the geometry of the bony part, the motion in the second one is primarily determined by the ligamentous structures (Offiah and Day, 2017). These two joints are responsible for the large mobility exhibited by the human cervical spine. While the changes in the geometry (curvature of the different sections of the spine), size and structure of the vertebrae and intervertebral discs during development and up to maturity are extensively reported in the literature (Moore et al., 2010), the effects of aging on the spine are limited to the overall decrease in bone density that modifies the geometry of the vertebral bodies and facilities the development of osteophytes around the attachment of the intervertebral discs to the bone. In parallel, osteophyte growth around the joint capsules is also possible and is normally associated to the wearing out of the cartilage with age. Osteophytes may occur at any level of the spine, including the atlanto-axial joint (Alikhani et al., 2020). The combination of the stiffening effect of the osteophytes and the degradation of the ligaments in the cervical spine with age has been suggested as a risk factor to the increased likelihood of upper cervical injuries (atlanto-axial junction, odontoid injuries) observed in elderly patients, as the lower cervical spine would become stiffer and transmit increased loads to the upper cervical spine region (Lomoschitz et al., 2002).

Subject-specific estimation of the inertial properties of the head of the volunteers is essential to obtain a good prediction of the loads calculated using inverse dynamics (Yoganandan et al., 2009; Seacrist et al., 2011; Beeman et al., 2016). Volunteer studies require non-invasive estimations of the head inertial properties, based on relationships of some external measurements taken on the volunteers (Loyd et al., 2010; Seacrist et al., 2011). This study combined data from several cadaveric studies that had measured the inertial properties of the head to propose new relationships to estimate the head mass and the moment of inertia of the head of the volunteers. Compared to previous estimations of these properties, the method developed here reduced the error of previous publications (achieving an estimated mean error of 11.4 ± 9.1% for the head mass and 13.9 ± 12.4% for the head moment of inertia). Nevertheless, previous studies (Seacrist et al., 2011) had used data from PMHS up to 16 years of age, which could explain the larger error obtained for adults. Compared to previous research, the results obtained for the head mass (4.20 ± 0.13 kg) and moment of inertia (0.0251 ± 0.0028 kg m^2^) were within the expected range reported in earlier studies (Beier et al., 1980; Plaga et al., 2005; Damon, 2009). The present study also showed that, for the volunteers included in this study, head inertial parameters were independent of age, but were dependent, as expected, on head dimensions.

The inverse dynamics method used in this study to estimate the upper neck loads assumes a pin joint between the head and the first cervical vertebra, which is an oversimplification of the real anatomy of the head-neck junction. The forces and moments estimated here are not supported by a single point anatomical structure, but are in fact distributed among the condyles of C1 and the cervical ligaments and muscles. It is also not possible to apportion the load that each of the anatomical structures would receive in case of a sudden deceleration. However, the pin joint model is closer to the construction of the ATD neck and can be used to inform more biofidelic designs of the dummy neck.

With the experimental data available for this study, it was difficult to find differences in the time history plots of the upper cervical forces estimated for the young and elderly age groups. The potential differences that could be attributed to age, are included in the variability observed in each of the groups, which is especially significant in the younger group. It can be observed that the values estimated for the shear (*F*_*x*_) and axial forces (*F*_*z*_) experimented by the elderly volunteers are within the highest values observed in the young group, although one young volunteer (Vol 05) exhibited larger force values than the ones observed in the elderly group. The situation is slightly different looking at the estimation of the flexion moment which is maximum for two of the elderly volunteers, supporting the anatomical/clinical observations mentioned above (Lomoschitz et al., 2002). It is important to mention that none of the volunteers complained of any cervical pain or even discomfort during the tests. Vol 08 was exposed only to two trials as he was experiencing discomfort from the rigid seat plate used in the tests.

In Mertz and Patrick (1971), one volunteer was exposed to 46 sled runs at various degrees of severity to induce neck flexion. This volunteer experienced pain in the neck and back after one sled run, and did not desire to go further. The peak acceleration for this run was 9.6 g, the maximum head accelerations observed were 573 *rad*/*s*^2^ in flexion and −760 *rad*/*s*^2^ in extension, the estimated peak moment was 90.7 Nm, and the peak axial and shear forces were 647.6 N and 789.6 N, the latter occurring 20 ms after the peak axial force. These values exceeded the ones observed in the volunteer tests presented here. The researchers proposed the value *My* = 90.7 Nm as the injury threshold for living humans. In PMHS tests performed under similar conditions but with increasing acceleration levels, Mertz and Patrick (1971) did not find any indication of disc, ligament or bony cervical injuries for values up to *My* = 189.8 Nm, although the authors advised caution to accept this level as muscular injury could have happened in a living human. Focusing on the volunteer sled runs that occurred at deceleration levels comparable to those of our study (2.9–4.2 g), the peak moment observed in Mertz and Patrick (1971) varied between 11.7 and 20.75 Nm and the shear force ranged between 160.1 and 280.2 N. These values are much closer to the ones observed in this study. It should be noted that the volunteer in the Mertz and Patrick (1971) was restrained using a crisscross seatbelt over his chest, resulting in an earlier rotation of the head that could explain the different timing for the peak values observed in the two studies. The authors proposed that the best indicator for the degree of severity of neck flexion is the equivalent moment at the occipital condyles.

However, the suggested injury threshold for the flexion moment My in Mertz and Patrick (1971) is higher than the threshold suggested in a later study (Prasad and Daniel, 1984), which found severe neck injuries in piglets starting at neck moment values of 29.4 Nm. However, the data in the latter study are difficult to translate to the case of humans due to the use of juvenile surrogates and to reporting neck values measured with a pediatric ATD instead of using inverse dynamics. As there was one case in which the piglet did not suffer any neck injury after being exposed to a moment of 50.9 Nm, Prasad and Daniel (1984) hypothesized that the mechanism of neck injury required the combined action of a flexion moment and an axial load. This hypothesis could be related to the finding of this study in which the peak axial force and the peak bending moment occurred at very similar timing.

If the focus is on low-speed frontal impacts, several contemporary studies have calculated the upper cervical forces and moments of volunteers using inverse dynamics (Arbogast et al., 2009; Lopez-Valdes et al., 2010; Seacrist et al., 2011; Beeman et al., 2016). Although the deceleration level used in these studies is similar, there were important differences in the experimental setup and in the initial position of the participants that affected the excursion of the head and the calculated neck loads (Beeman et al., 2016). The younger volunteer group in this study matches closely the 18–30 years old group studied in Seacrist et al. (2011). The latter reported mean peak values of −162 ± 24*N* and 13 ± 2.7*Nm* for the axial force and bending moment, which are very close to the ones included here.

The values of the forces and moments obtained can be compared also to those reported by Funk et al. (2011) during everyday vigorous activities. The 20 volunteers included in this study spanned a range of age between 26 and 58 years and the results also showed a large variability between the peak values of the shear and axial force, and of the flexion moment measured during the tests. In general the shear forces measured in Funk et al. (2011) were smaller than the ones calculated in this study, while the axial forces peak values were larger. The estimated My flexion moment was comparable especially for some of the daily activities that occurred at a higher rate such as shaking the head (15 ± 5.7*Nm*), or being dropped while seating supine in a chair (15 ± 5.7*Nm*). Funk et al. (2011) did not find any effect of age and body size of the volunteers on the biomechanical measurements or symptoms being reported in any of the test scenarios.

There are some limitations of the study that need to be discussed. First, each volunteer was exposed to several trials (between two and five), but the results included here correspond to only one trial per volunteer. Volunteers were asked to remain relaxed during the simulated impact, but it must be assumed some level of reflex muscle contraction, which could have influenced the calculated loads (Beeman et al., 2016). To minimize the influence of this non-voluntary muscle response, and after performing a preliminary analysis that found differences in the kinematics between the first trial and the remaining ones for each volunteer, the third trial was the one used in the study (with the exception of two volunteers as discussed in the Methods section). Second, there were only four volunteers in the elderly group. Even if the recruitment period was open for several weeks, it was difficult to secure more volunteers willing to participate in the study. Despite of it, other studies have used groups with 5–6 subjects in similar analyses (Arbogast et al., 2009; Beeman et al., 2016). Third, as the sample size was limited, it was decided to avoid averaging the responses of the volunteers so that individual differences among subjects could be appreciated. This decision implies that detecting the potential differences between the two age groups could have become more difficult, but respects the nature of the individual data.

As indicated in Table 2, the analyses of the kinematics of the volunteers required the differentiation of instrument data. These procedures usually involved the amplification of the noise in the signals that had to be filtered before calculating the estimated values of the neck loads, similarly to what had been reported in previous studies (Funk et al., 2007; Lopez-Valdes et al., 2010; Seacrist et al., 2011). In our case, as the head instrumentation was fixed to the head using a head band that did not provide a perfectly rigid attachment to the head, some of the experimental data required to be filtered before being able to process them. Since the rigidity of the head band attachment changed between the volunteers, different cutoff frequencies were used. To minimize the impact of the filtering on the original data, the cutoff frequency for the filters was selected after visual inspection of the original (unfiltered) and processed signals, together with the analysis of the frequency content of the original signal using the Fast Fourier Transform of the experimental data.

## 5. Conclusion

Using previously obtained data from PMHS, this study proposed new relationships to calculate the inertial properties of the human head that improved substantially the methods that had been used in previous literature. These relationships were used then in the estimation of the axial and shear force, and the sagittal moment experienced by volunteers at the craniocervical junction during low-speed frontal decelerations (9 km/h). Two groups of volunteers were analyzed: a young adult group (18–30 years old) and an elderly group (>65 years old). Although slightly greater values of the peak My moment were found in the elderly group, they were within the variability observed in the young group. Thus, with the limited sample analyzed in this study, no substantial differences were found in the comparison of craniocervical loads between the two age groups. The results reported here can be used to benchmark active human body models in low-speed frontal impacts. The findings of this study support that the active response of the cervical spine of human body models does not need to account for age effects in the adulthood.

## Data Availability Statement

The data analyzed in this study is subject to the following licenses/restrictions: Data of volunteers available on request. Requests to access these datasets should be directed to steffen.peldschus@med.uni-muenchen.de.

## Ethics Statement

The studies involving human participants were reviewed and approved by CEICA (Ethical Comission for Clinical Research of Aragon). The patients/participants provided their written informed consent to participate in this study.

## Author Contributions

CV-T: data analyses, methodology, and manuscript writing. MV: data analyses, methodology, and manuscript review. JJ-O: conceptualization, supervision, and manuscript review. JM: conceptualization, experimental work, and manuscript review. SS: conceptualization, methodology, and manuscript review. SP: conceptualization, resources, methodology, and manuscript review. FL-V: conceptualization, experimental work, methodology, supervision, and manuscript review. All authors contributed to the article and approved the submitted version.

## Conflict of Interest

The authors declare that the research was conducted in the absence of any commercial or financial relationships that could be construed as a potential conflict of interest.

## References

[B2] Albery C. Whitestone J. (2003). Comparison of cadaveric human head mass properties: mechanical measurement vs. calculation from medical imaging, in Proceedings of the 31st International Workshop Injury Biomechanics Research, Vol. 157, 157–172.

[B3] Alikhani P. Suradi Y. Amin S. Amin U. (2020). Complex c1-2 osteophyte presenting with severe dysphagia and ptosis. Neurology 94, 324–325. 10.1212/WNL.0000000000008969 31964690

[B4] Arbogast K. Balasubramanian S. Seacrist T. Maltese M. García-España J. Hopely T.. (2009). Comparison of kinematic responses of the head and spine for children and adults in low-speed frontal sled tests. Stap Car. Crash J. 53, 329–732. 10.4271/2009-22-0012 20058560

[B5] Beeman S. M. Kemper A. R. Duma S. M. (2016). Neck forces and moments of human volunteers and post mortem human surrogates in low-speed frontal sled tests. Traffic Inj. Prev. 17(Suppl. 1):141–149. 10.1080/15389588.2016.1205190 27586115

[B6] Beier G. Schuller E. Schuck M. Ewing C. L. Becker E. D. Thomas D. J. (1980). Center of gravity and moments of inertia of human heads, in Proceedings of the International Research Council on Biomechanics of Injury (IRCOBI), (Birmingham).

[B7] Clauser C. E. McConville J. T. Young J. W. (1969). Weight, Volume, and Center of Mass of Segments of the Human Body. Technical Report AMRL-TR-69-70, Air Force System Comand, Wright-patteron AFB.

[B8] Damon A. (2009). Characterizing the Geometric and Inertial Properties of the Adult Human Head. Master's Thesis.

[B9] Eppinger R. Kuppa S. Saul R. Sun E. (2000). Supplement: Development of Improved Injury Criteria for the Assessment of Advanced Automotive Restraint Systems II. Technical report, United States. National Highway Traffic Safety Administration.

[B10] Funk J. Cormier J. Bain C. Guzman H. Bonugli E. (2007). An evaluation of various neck injury criteria in vigorous activities, in Proceedings of the International Research Council on Biomechanics of Injury (IRCOBI) (Maastricht), 19–21.

[B11] Funk J. Cormier J. Bain C. Guzman H. Bonugli E. Manoogian S. (2011). Head and neck loading in everyday and vigorous activities. Ann. Biomed. Eng. 39, 766–776. 10.1007/s10439-010-0183-3 20960061

[B12] Li F. Liu N.-S. Li H.-G. Zhang B. Tian S.-W. Tan M.-G.. (2019). A review of neck injury and protection in vehicle accidents. Transport. Safety Environ. 1, 89–105. 10.1093/tse/tdz012

[B13] Lomoschitz F. Blackmore C. Mirza S. Mann F. (2002). Cervical spine injuries in patients 65 years old and older: epidemiologic analysis regarding the effects of age and injury mechanism on distribution, type, and stability of injuries. Am. J. Roentgenol. 178, 573–577. 10.2214/ajr.178.3.1780573 11856676

[B14] López-Valdés F. J. Juste-Lorente O. Maza-Frechin M. Pipkorn B. Sunnevang C. Lorente A.. (2016). Analysis of occupant kinematics and dynamics in nearside oblique impacts. Traffic Inj. Prev. 17(Suppl. 1):86–92. 10.1080/15389588.2016.1189077 27586108

[B15] Lopez-Valdes F. J. Lau A. Lamp J. Riley P. Lessley D. J. Damon A.. (2010). Analysis of spinal motion and loads during frontal impacts. Comparison between pmhs and atd. Ann. Adv. Autom. Med. 54, 61–78. 21050592 PMC3242563

[B16] Lopez-Valdes F. J. Mroz K. Eggers A. Pipkorn B. Muehlbauer J. Schick S.. (2018). Chest injuries of elderly postmortem human surrogates (pmhss) under seat belt and airbag loading in frontal sled impacts: comparison to matching thor tests. Traffic Inj. Prev. 19(Suppl. 2):S55–S63. 10.1080/15389588.2018.1542139 30543304

[B17] Loyd A. Nightingale R. Bass C. Mertz H. Frush D. Daniel C.. (2010). Pediatric head contours and inertial properties for atd design. Stapp Car Crash J. 54, 167–196. 10.4271/2010-22-0009 21512908

[B18] McConville J. T. Clauser C. E. Churchill T. D. Cuzzi J. Kaleps I. (1980). Anthropometric Relationships of Body and Body Segment Moments of Inertia. Technical Report AFAMRL-TR-80-119, Air Force System Comand, Wright-patteron AFB.

[B19] Mertz H. J. Irwin A. L. Prasad P. (2003). Biomechanical and Scaling Bases for Frontal and Side Impact Injury Assessment Reference Values. Technical report, SAE Technical Paper. 17096249 10.4271/2003-22-0009

[B20] Mertz H. J. Patrick L. M. (1971). Strength and response of the human neck, in Proceedings of the 15th Stapp Car Cash Conference (Coronado, CA), SAE paper No. 710855:207–255.

[B21] Moore K. Dalley A. Agur A. (2010). Clinically Oriented Anatomy. Clinically Oriented Anatomy. Philadelphia, PA: Wolters Kluwer Health/Lippincott Williams & Wilkins.

[B22] Muehlbauer J. Schick S. Draper D. Lopez-Valdes F. J. Symeonidis I. Peldschus S. (2019). Feasibility study of a safe sled environment for reclined frontal deceleration tests with human volunteers. Traffic Inj. Prev. 20(Suppl. 2):S171–S174. 10.1080/15389588.2019.1659592 31674808

[B23] Offiah C. E. Day E. (2017). The craniocervical junction: embryology, anatomy, biomechanics and imaging in blunt trauma. Insights Into Imaging 8, 29–47. 10.1007/s13244-016-0530-5 27815845 PMC5265194

[B24] Parenteau C. S. Viano D. C. (2014). Spinal fracture-dislocations and spinal cord injuries in motor vehicle crashes. Traffic Inj. Prev. 15, 694–700. 10.1080/15389588.2013.867434 24433030

[B25] Pipkorn B. López-Valdés F. J. Juste-Lorente O. Insausti R. Lundgren C. Sunnevång C. (2016a). Assessment of an innovative seat belt with independent control of the shoulder and lap portions using thor tests, the thums model, and pmhs tests. Traffic Inj. Prev. 17(Suppl. 1):124–130. 10.1080/15389588.2016.1201204 27586113

[B26] Pipkorn B. Sunnevang C. Juste-Lorente O. Maza M. Lopez-Valdes F. (2016b). Exploratory study of the kinematics of the thor dummy in nearside oblique impacts, in Proceedings of the International Research Council on Biomechanics of Injury (IRCOBI) (Malaga).

[B27] Plaga J. A. Albery C. Boehmer M. Goodyear C. (2005). Design and Development of Anthropometrically Correct Head Forms for Joint Strike Fighter Ejection Seat Testing. Wright-Pattersons AFB, OH.

[B28] Prasad P. Daniel R. P. (1984). A Biomechanical Analysis of Head, Neck, and Torso Injuries to Child Surrogates Due To Sudden Torso Acceleration. SAE Transactions 784–799.

[B1] SAE (2007). Instrumentation for Impact Test: Part 1-Electronic Instrumentation. Technical Report, SAE J211/1.

[B29] Safiri S. Kolahi A.-A. Hoy D. Buchbinder R. Mansournia M. A. Bettampadi D.. (2020). Global, regional, and national burden of neck pain in the general population, 1990-2017: systematic analysis of the global burden of disease study 2017. BMJ 368:m791. 10.1136/bmj.m791 32217608 PMC7249252

[B30] Seacrist T. Arbogast K. B. Maltese M. R. García-Espaňa J. F. Lopez-Valdes F. J. Kent R. W.. (2011). Kinetics of the cervical spine in pediatric and adult volunteers during low speed frontal impacts. J. Biomech. 45, 99–106. 10.1016/j.jbiomech.2011.09.016 22056197

[B31] THUMSUserCommnunity (2021). Seniors Deliverable 2.3. Available online at: https://tuc-project.org/frontal-sled-seniors/ (accessed May 14, 2021).

[B32] Umana E. Khan K. Baig M. Binchy J. (2018). Epidemiology and characteristics of cervical spine injury in patients presenting to a regional emergency department. Cureus 10::e2179. 10.7759/cureus.2179 29651372 PMC5893180

[B33] Walker L. Harris E. Pontius U. (1973). Mass, volume, center of mass, and mass moment of inertia of head and head and neck of human body, in Proceedings of the Stapp Car Crash Conference (Oklahoma City, OK), 525–538.

[B34] Yadollahi M. Paydar S. Ghaem H. Ghorbani M. Mousavi S. M. Taheri Akerdi A.. (2016). Epidemiology of cervical spine fractures. Trauma Mon. 21:e33608. 10.5812/traumamon.33608 27921020 PMC5124335

[B35] Yoganandan N. Maiman D. J. Guan Y. Pintar F. (2009). Importance of physical properties of the human head on head-neck injury metrics. Traffic Inj. Prev. 10, 488–496. 10.1080/15389580903132801 19746313

